# New Physical Hydrogels Based on Co-Assembling of FMOC–Amino Acids

**DOI:** 10.3390/gels7040208

**Published:** 2021-11-12

**Authors:** Alexandra Croitoriu, Loredana E. Nita, Aurica P. Chiriac, Alina G. Rusu, Maria Bercea

**Affiliations:** “Petru Poni” Institute of Macromolecular Chemistry, 41-A Grigore Ghica Voda Alley, 700487 Iasi, Romania; croitoriu.alexandra@icmpp.ro (A.C.); lnazarie@yahoo.co.uk (L.E.N.); achiriac@icmpp.ro (A.P.C.); bercea@icmpp.ro (M.B.)

**Keywords:** supramolecular gels, amino acids, co-assembly, low molecular weight gelators

## Abstract

In the last years, physical hydrogels have been widely studied due to the characteristics of these structures, respectively the non-covalent interactions and the absence of other necessary components for the cross-linking processes. Low molecular weight gelators are a class of small molecules which form higher ordered structures through hydrogen bonding and π–π interactions. In this context it is known that the formation of hydrogels based on FMOC–amino acids is determined by the primary structures of amino acids and the secondary structure arrangement (alpha–helix or beta–sheet motifs). The present study aimed to obtain supramolecular gels through co-assembly phenomenon using FMOC–amino acids as low molecular weight gelators. The stability of the new structures was evaluated by the vial inversion test, while FTIR spectra put into evidence the interaction between the compounds. The gel-like structure is evidenced by viscoelastic parameters in oscillatory shear conditions. SEM microscopy was used to obtain the visual insight into the morphology of the physical hydrogel network while DLS measurements highlighted the sol-gel transition. The molecular arrangement of gels was determined by circular dichroism, fluorescence and UV-Vis spectroscopy.

## 1. Introduction

Supramolecular chemistry includes and investigates highly ordered systems formed through intra- and intermolecular combination by weaker and reversible non-covalent interactions, and, also, it studies the recognition and selection of molecular components found in and for these structures [1]. Low molecular weight gelators (LMWGs) are a class of compounds with small dimensions (<3 kDa) [2] that present the property of self-association by physical interactions (hydrogen bonds, ionic, hydrophobic, van der Waals, π–π stacking) in a three-dimensional network, and have the ability to incorporate solvent molecules into their structure [3]. The peptides and amino acids are organic compounds that can be included in the category of low molecular weight gelators. Tryptophan (Trp) is an essential amino acid, precursor of serotonin and tryptamine neurotransmitters [4] and likewise of epiphyseal hormone melatonin [5]. As an essential component of proteins, tryptophan is involved in multiple physiological processes, being a key factor in the regulation of circadian rhythm and pain perception. Tryptophan levels from blood circulation influence the availability of serotonin having effects in psycho-neural control, by inhibiting the presynaptic hippocampal cholinergic terminals [6,7,8].

From a chemical point of view, tryptophan is an aromatic polar amino acid with hydrophobic character, which has an indole ring in the structure.

Another amino acid used for the production of antiseptic materials, due to its antibacterial activity against Gram-positive bacteria, and applied in the biomedical field is lysine (Lys), which is a polar α–amino acid with hydrophilic character.

Generally, the short peptides and amino acids are modified at the N–terminal with a voluminous aromatic group, such as 9–fluorenylmethyloxycarbonyl (FMOC), naphthalene, anthracene, carbazole or tert–butoxycarbonyl (BOC). The FMOC group is offering a base-sensitive protection group, while its aromatic rings have remarkable physicochemical properties in the formation of organized structures through π–π stacking [9].

Another investigation in the field underlined that the perfect equilibrium between hydrophilic and hydrophobic moieties is one of the key factors in obtaining self-assembled supramolecular gels [10].

The gelling process can be triggered by external factors such as pH changes, temperature, solvent polarity and variation of ionic strength [11]. These factors lead to a variety of secondary structural arrangements like β–sheet, β–hairpin and α–helix, and are dependent on the intra and intermolecular bonds formed between amino acids residues. The π–π interactions occurring between the aromatic moieties of two peptides have a vital role in the self-assembly processes, resulting in β–sheet structures [12].

The supramolecular gels based on peptides and amino acids present particular importance in biomedical applications due to intrinsic remarkable properties. This class of soft materials is utilized especially in drug delivery, [13] wound dressing with antibacterial action and repairing effect [14] or 3D support for the development of the cell culture [9]. The peptides are considered attractive for the biomedical fields due to the mimetic biochemical processes of the natural extracellular matrices (ECM), nanofibrous aspects and high hydration. The main advantages of these small molecules are their biocompatibility, bioactivity, cellular adhesion and proliferation. These features are essential for the development of the three-dimensional structures with role in cellular growth [15].

Even if various studies presented in the literature [16] have aimed to understand the peptide self-assembly processes, it has not yet been fully possible to conceive how molecules can self-organize.

In this context, the present study aims to obtain and characterize co-assembled supramolecular hydrogels with lysine and tryptophan content. The gelling process was realized through pH changes and using a polar solvent.

## 2. Results and Discussion

### 2.1. Self-Assembling Process

**Figure 1:**
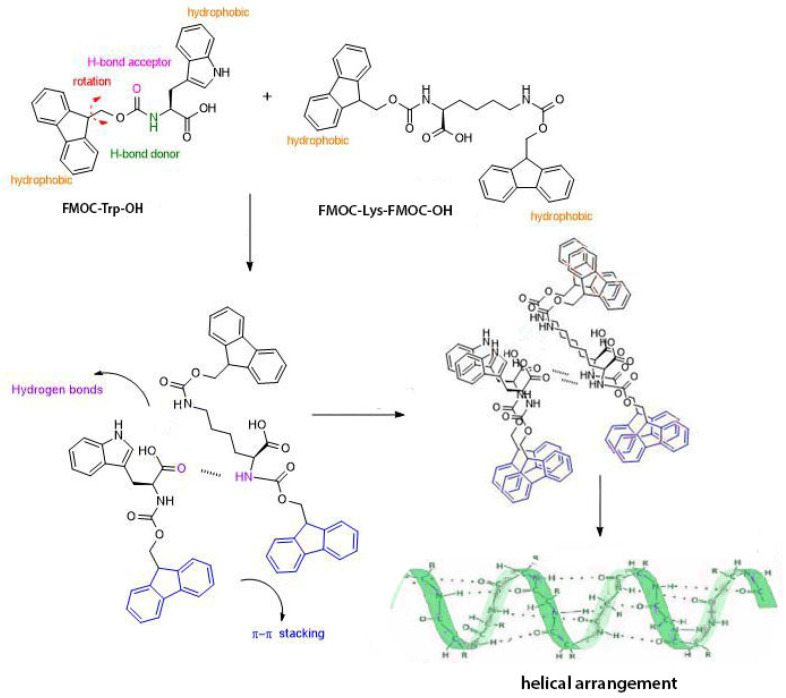
The bonds involved in the formation of the primary structure and the illustration of helical molecular arrangement according to molecular arrangement results (adapted from [18,19]).

The use of LMWGs in co-assembled structures is guided by the intention for developing new materials with superior properties. Thus, the mixing of two different components can lead to the formation of a network that combine mechanical and biological properties of both. The advantage of these small molecules is given by their property to self-associate and form fibrous, tubular or helical structures through non-covalent interactions. The forces involved in the formation of primary structure, respectively secondary structure are hydrogen bonds, Van der Waals forces, π–π stacking or other physical forces [17]. The amino acids used in this study, FMOC–Trp–OH and FMOC–Lys–FMOC–OH can self-assemble in ordered structures by forming of a supramolecular three-dimensional network that can encapsulate high water content (Figure 1). The gelling process is triggered by pH changes in the case of FMOC–Trp, and is influenced by solvent polarity in the case of FMOC–Lys–FMOC–OH. The β–sheet molecular arrangements occur when peptide side chains contain both hydrophilic and/or hydrophobic groups which promote the formation of intermolecular interactions.

**Figure 2:**
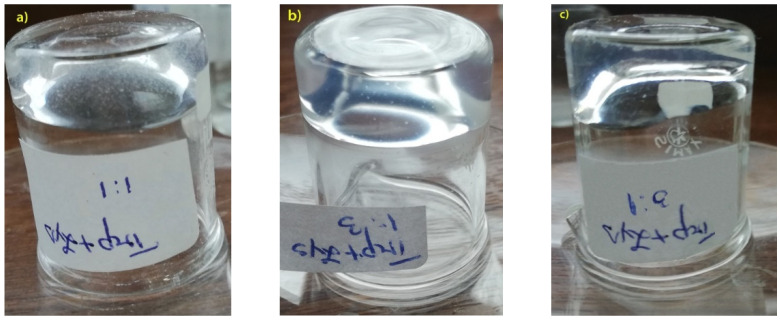
Self-supporting gels based on amino acids: (**a**) FMOC_Lys_FMOC–OH FMOC–Trp_FMOC–Lys–FMOC_1/1, (**b**) FMOC–Trp_FMOC–Lys–FMOC_1/3, (**c**) FMOC–Trp_FMOC–Lys–FMOC_3/1.

The gels obtained through co-assembly processes between the two functional amino acids with an aromatic group (FMOC) on the N–terminal have a stable structure confirmed by the vial inversion test (Figure 2). Additionally, self-supporting gels have a transparent aspect following the co-assembly process. The FMOC–Trp_FMOC–Lys–FMOC sample in 1/3 ratio presents viscoelastic behavior and has a translucent aspect, while the FMOC–Trp_FMOC–Lys–FMOC sample in 3/1 ratio is slightly unstable, which was attributed to the lower amount of lysine. In all three cases stable gels were obtained that have passed the vial inversion test (Figure 2).

#### 2.1.1. Dynamic Light Scattering

**Figure 3:**
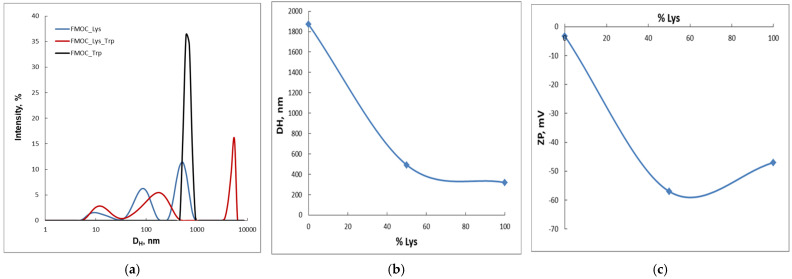
(**a**) Particle size distribution of FMOC_Lys/FMOC_Trp = 1/1 sample compared to FMOC–Lys–FMOC–OH and FMOC_Trp–OH; (**b**) hydrodynamic diameter (DH), (**c**) zeta potential (ZP) as function of the lysine amount.

DLS studies (Figure 3a,b) show the presence of large particles (approx. 1800 nm) and higher polydispersity (PDI = 0.8) in the case of FMOC–Trp–OH. The FMOC–Lys–FMOC–OH sample presents smaller trimodal distribution (320 nm) and better PDI (0.5). In the case of the co-assembly complex between the two investigated amino acids, a bimodal distribution is obtained, with dimensions closer to FMOC_Lys–FMOC–OH (490 nm) and a better polydispersity (PDI = 0.3). This sustains the formation of intermolecular interactions between the co-partners and the organization in a supramolecular structure. In fact, the peak at 5120 nm that is recorded in the case of the complex indicates the beginning of the aggregation and the three-dimensional structuring with the formation of the supramolecular gel. Regarding the value of the zeta potential (Figure 3c), in the case of the supramolecular complex, a higher value of the zeta potential modulus is registered comparative to the starting components. Thus, the FMOC–Lys–FMOC–OH/Fmoc–Trp–OH = 1:1 sample has the zeta potential of −57 mV compared to the starting compounds that have −3.26 mV (FMOC–Trp–OH) and −47 mV (FMOC–Lys–FMOC–OH), behavior that attests to the stability of the system. This behavior confirms the interactions occurring between the compounds.

#### 2.1.2. Molecular Arrangement

**Figure 4:**
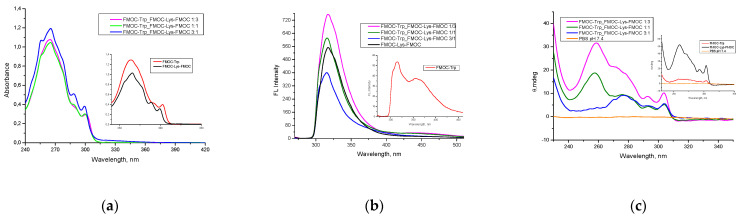
(**a**) UV-Vis absorbance, (**b**) fluorescence and (**c**) dichroism spectra of the hydrogels.

In Figure 4a are reported the UV-Vis spectroscopic results for FMOC–Trp_FMOC–Lys–FMOC: 1/3, FMOC–Trp_FMOC–Lys–FMOC: 1/1 and FMOC–Trp_FMOC–Lys–FMOC: 3/1 co-assembled hydrogels, respectively the simple hydrogels like FMOC–Trp–OH and FMOC–Lys–FMOC–OH (Figure 4—inset). The UV-Vis absorbance spectra of co-assembled hydrogels showed a dominant peak at 265 nm with two shoulders at 288 and 300 nm. According to the study realized by S. Prasad et al. [20], the absorption features seen around the 255–280 nm region are assigned to chromophores present in the side chains of the aromatic amino acids. In the case of Trp, the indole ring is considered the mainly source of UV absorbance and emission and increased the intensity of the adsorption bands [20,21]. Thus, FMOC–Trp–OH and FMOC–Trp_FMOC–Lys–FMOC:3/1 present a higher intensity of the absorption bands comparative to the other sample where the amount of FMOC–Trp–OH decreases.

The aromatic amino acids are molecules with inherent fluorescence while the aliphatic amino acids require the addition of fluorescent moieties [22,23]. The corresponding fluorescence spectra of the obtained hydrogels are shown in Figure 3b. The fluorescence spectra of the FMOC–Lys–FMOC–OH show a ℷmax at 317 nm, while FMOC–Trp (Figure 4b—inset) has ℷmax at 314 nm with one broad shoulder at 355 nm. These characteristic peaks indicate the presence of FMOC groups and Trp residues [8]. As shown in Figure 4b, the co-assembled hydrogels show a similar ℷmax at 315 nm, but different peaks intensity. The volumetric ratio between the componds influences the fluorescence intensity. The amount of FMOC–Lys–FMOC–OH from the hydrogels is directly proportional to the fluorescence intensity. Indeed, an increase in the amount of FMOC–Lys–FMOC–OH leads to a change in the fluorescence intensity and may be attributed to a higher number of available fluorophore moieties [8]. According to the literature data [24], the increase in peaks intensity may suggest the formation of aromatic interaction between fluorenyl groups or π–π overlapping interactions.

Circular dichroism spectroscopy is a technique which provides useful information about the secondary structures of proteins from a number of spectral regions [25]. According to the study realized by Sharon M. Kelly et al. [26], the peptide bond, aromatic amino acid side chains and disulfide bonds are considered the chromophores of interest for CD studies of proteins. The characteristic absorption band of peptide bond is situated below 250 nm, while absorption bands in the region 260 to 300 nm are assigned to the aromatic amino acid side chains [27]. Likewise, the disulfide bonds absorption band is characterized by a weak broad absorption band centered around 260 nm.

The CD results (Figure 4c) for FMOC-based amino acid gels show that co-assembled gels exhibited an induced CD with all positive peaks around 240–320 nm. According to the literature data [28], all positive peaks on the CD spectra might suggest a unique supramolecular organization of FMOC moieties into highly ordered 2D self-assembled structures. As shown in Figure 3c inset and aforementioned (in Figure 3a and Figure 4b), the volumetric ratio between FMOC–Trp–OH and FMOC–Lys–FMOC–OH influences the aspect of the co-assembled hydrogels spectra. The FMOC–Trp_FMOC–Lys–FMOC 1/3 and FMOC–Trp_FMOC–Lys–FMOC 1/1 samples present a strong positive peak at 258 nm, which does not appear at the FMOC–Trp_FMOC–Lys–FMOC 3/1 sample. The main peak for the FMOC–Trp_FMOC–Lys–FMOC 3/1 sample is around 275 nm. Other peaks present in the CD spectra are at 290 nm, respectively 305 nm. The absorption bands in the region of 250–290 nm are associated with the presence of fluorenyl moieties, while the absorption band of peak at 304 nm demonstrates that the supramolecular self-assembly was influenced by aromatic π–π stacking and stabilized by hydrogen bonding between –CO–NH groups [29].

The results obtained from CD spectroscopy corroborated with literature data [24,29,30] indicate a possible helical twist structural arrangement of co-assembled supramolecular hydrogels.

### 2.2. Characterization of Freeze-Dried Gels Obtained from the Self-Assembling Process

#### 2.2.1. FT-IR Analysis

**Figure 5:**
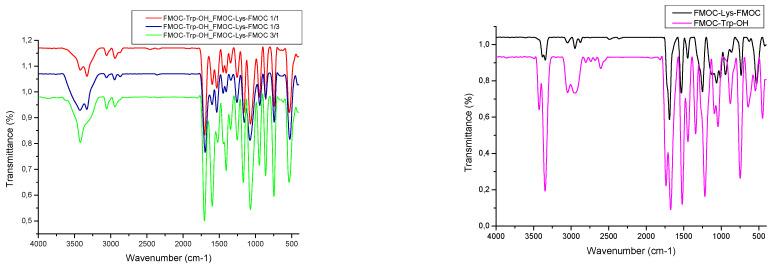
IR spectra of the synthesized amino acids-based hydrogels.

FT-IR spectroscopy (Figure 5) provides useful information about the changes which occur in the characteristic frequency bands of the new polymer structures. Table 1 summarizes important group frequencies for amino acids-based hydrogels.

The wide bands from the 3400 cm^−1^ region are characteristic of the presence of OH groups from water residues cached into the structure of freeze-dried gels. The C–H groups have the corresponding peak from 3046 cm^−1^ in case of FMOC–Lys–FMOC–OH and FMOC–Trp–OH spectra, which is moved to about 3055 cm^−1^ for freeze-dried gels, showing an interaction between them.

Specific stretching vibrations of the C=O from the carboxyl group is present at 1700–1690 cm^−1^ and provide the information on the formation of the hydrogen bonds. Therefore, the movement of the absorption bands from 1695 cm^−1^ (for FMOC–Trp_FMOC–Lys–FMOC: 1/3) to 1705 cm^−1^ (for FMOC–Trp_FMOC–Lys–FMOC: 3/1) indicates that a higher amount of FMOC–Lys–FMOC leads to the involvement of a higher amount of the carboxyl groups in the formation of intra and intermolecular hydrogen bonds.

The NH bond from the amide group appears for all samples analyzed, in the region 1530 cm^−1^, except for the FMOC–Trp_FMOC–Lys–FMOC: 3/1 sample which shows a displacement at 1522 cm^−1^, characteristic of the formation of hydrogen bonds.

The presence of bands in the region 1200–1300 cm^−1^ indicates C–O and C–N stretching vibrations. Their decrease in intensity with increasing of the FMOC–Trp–OH amount is due to a possible molecular reorientation of Trp molecules that participate in the formation of the gel. The absorption bands in the region 950–730 cm^−1^ are assigned to the specific groups of the aromatic rings from the FMOC structure, respectively the indole ring present in the Trp structure.

#### 2.2.2. TG/DTA Analysis

Table 2 presents the main parameters of the thermal decomposition of the synthesized gels. According to the literature data [31], the α–amino acids used in the obtaining of the freeze-dried hydrogels (FMOC–Trp–OH and FMOC–Lys–FMOC–OH) have high melting points due to the bonds of hydrogen between COO– and NH_3_^+^ groups from the common C atom.

**Figure 6:**
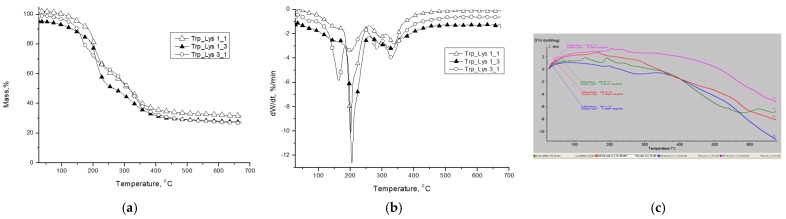
TG (**a**), DTG (**b**) and inflection point (Tg) from DTA curves of amino acids-based lyophilized gels. (**c**) DTA curves.

In Figure 6, the TG with respective DTG curves for the freeze-dried gels, with different volumetric ratio between the compounds, are presented. The samples FMOC–Trp_FMOC–Lys–FMOC in 1/1 ratio and FMOC–Trp_FMOC–Lys–FMOC in 3/1 ratio present four stages of thermal decomposition, while the FMOC–Trp_FMOC–Lys–FMOC variant in 1/3 ratio has just three stages. The differences in the thermal behavior of the analyzed gels can be corroborated with the volumetric ratio between the amino acids from the gel structure. Additionally, from the DTA curves (Figure 6c), the inflection point (Tg—Table 2) can be calculated. Comparative with FMOC_Lys_FMOC, which present a Tg at 84 °C, the presence of FMOC_Trp determined a decreased of Tg, this being included in the interval 30–48 °C depending on the ratio between the two components.

Thus, the decrease of the amount of lysine into the gels structure determined a decrease into the thermal stability. The FMOC–Trp_FMOC–Lys–FMOC sample in 1/3 ratio shows a significant weight loss (more than 46 wt.%) in the first two stages of thermal decomposition, at temperature below 250 °C. Nevertheless, the sample FMOC–Trp_FMOC–Lys–FMOC in 1/3 ratio presents the highest thermal stability according to T_10_ and T_20_ parameters (Table 2). The mass loss in the first two stages can be attributed to the break of the links from the N–terminal and aliphatic chains and the release of small compounds that form as a result of the thermal decomposition. This observation is in accordance with the study realized by Ehlers et al. [32] that sustains that the removal of the water molecules from the ammonium salt or the amide terminal group, at low temperature leads to the formation of carbodiimides. Generally, the first process presents a T_peak_ up to 165 °C, followed by the second process with a T_peak_ around 200 ± 5 °C. According to the literature data [33], the decomposition of carbamate group from FMOC structure at low temperature (below 200 °C) is influenced by the presence of aprotic dipolar solvents such as DMSO (which was used in the first stage of preparation of stock solution—according to the experimental part). For the third process, the T_peak_ was registered around 285 °C in the case of the samples with 1/1 and 3/1 molar ratio, and the correspondingly weight losses were about 24 wt.% and 12 wt.%. The research studies indicated that the degradation of the aromatic rings occur at higher temperatures due to the double bonds from their structure, in comparison with the aliphatic chains. According to the study of Makino [34], the decomposition of the aromatic rings (benzene and toluene) starts at a high temperature in the range of 350–500 °C, with the formation of intermediate structures (ethylene, butane, propene, butadiene, etc.) and the elimination of H_2_O and CO_2_. Therefore, it can be concluded that the last process with a T_peak_ at 330 °C corresponds to the aromatic rings decomposition from the FMOC structure, respective to the indole ring of tryptophan. The increased weight loss occurring in this stage in the case of the FMOC–Trp_FMOC–Lys–FMOC sample with 3/1 molar ratio (approximately 28 wt.%) is due to a higher content of the aromatic rings of the tryptophan structure. The residue, 29 wt.% in case of FMOC–Trp_FMOC–Lys–FMOC in 1/1 ratio, around 32 wt.% for FMOC–Trp_FMOC–Lys–FMOC in 1/3 ratio and 27 wt.% for FMOC–Trp_FMOC–Lys–FMOC in 3/1 ratio, is attributed to an incomplete decomposition of fluorene rings from the FMOC structure.

### 2.3. Viscoelastic Behavior

**Figure 7:**
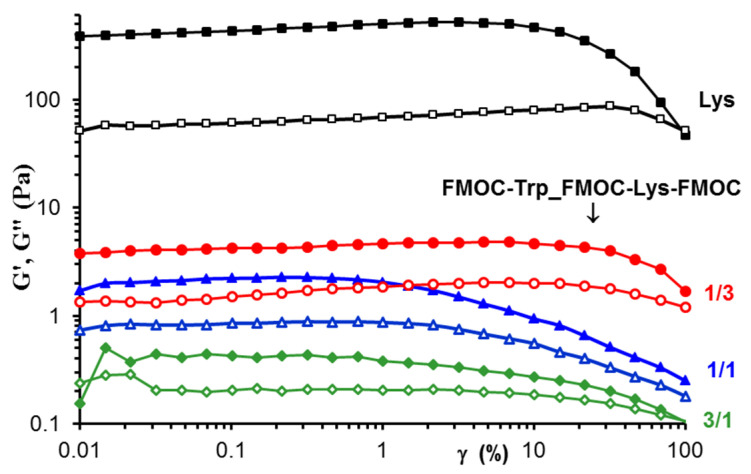
The viscoelastic moduli as a function of deformation at 25 °C and ω = 1 rad/s: G’—full symbols; G′′—open symbols.

Figure 7 presents the viscoelastic moduli, G′ and G′′, determined as a function of deformation (γ) in amplitude sweep tests at constant oscillation frequency (ω) of 1 rad/s. All samples, Lys and FMOC–Trp_FMOC–Lys–FMOC of different compositions present gel-like behavior with G′ > G′′. The linear range of viscoelasticity is influenced by the sample composition, being extended for the Lys sample and FMOC–Trp_FMOC–Lys–FMOC in 1/3 ratio, when the nonlinear behavior starts for γ > 10%. For FMOC–Trp_FMOC–Lys–FMOC in 1/1 and 3/1 ratios, the linear behavior is observed below γ = 1%.

**Figure 8:**
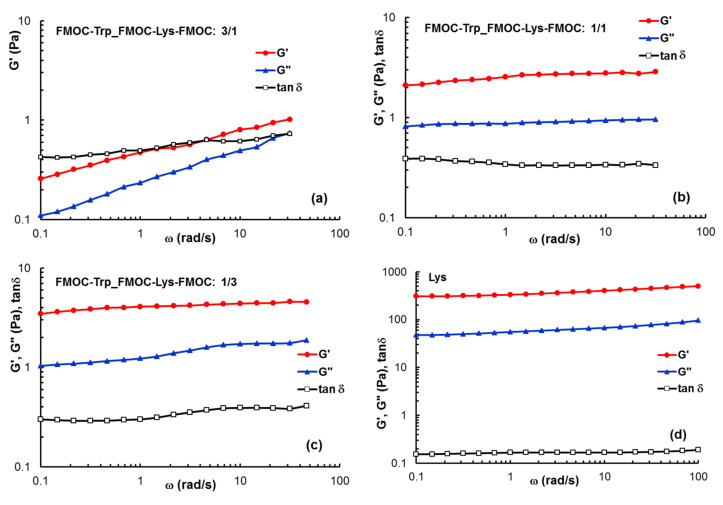
The dependences of G’, G”and tan δ as a function of the oscillation frequency at 25 °C and γ = 1% for the investigated gel samples: (**a**) FMOC–Trp_FMOC–Lys–FMOC: 3/1; (**b**) FMOC–Trp_FMOC–Lys–FMOC: 1/1; (**c**) FMOC–Trp_FMOC–Lys–FMOC: 1/3; (**d**) Lys.

According to the data from Figure 6, the value of 0.3% was selected for the frequency sweep tests, when for all samples the linear range of viscoelasticity is registered. Figure 7 gives the dependences of G’, G”and tan δ as a function of ω for all FMOC–Trp_FMOC–Lys–FMOC samples (Figure 8a–c), as compared with Lys (Figure 8d). The gel−like behavior (G′ and G′′ independent of ω, G′ > G′′ and tan δ < 1) is observed for Lys and samples with high Lys content: FMOC–Trp_FMOC–Lys–FMOC: 1/3 and 3/1. The viscoelastic moduli decreases as FMOC–Trp content increases and for FMOC–Trp_FMOC–Lys–FMOC: 3/1 a weak gel is obtained: G′ > G′′ and tan δ < 1, but G’~ω^0.23^ and G”~ω^0.33^.

The values of G’ are correlated with the network strength [35,36,37]. From Figure 5 and Figure 6 it can be observed that Lys content in the sample influences considerably the gel strength.

### 2.4. SEM Microscopy

**Figure 9:**
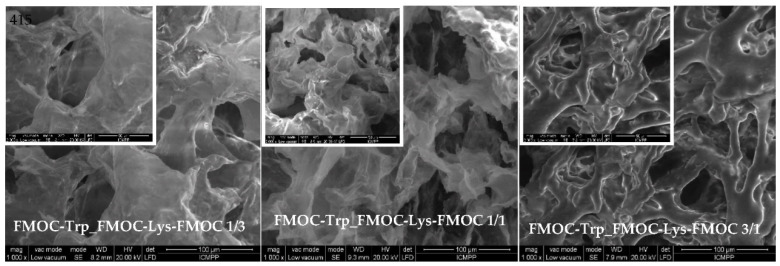
SEM pictures of amino acids-based lyophilized gels at 1000× magnification. Inset: The magnification of picture was 2000×.

Hydrogels are three-dimensional networks with substantial water content. The integrity of the hydrogel changes with the removal of water from the system. The morphological characterization of hydrogels by SEM analysis provides essential information on the size, shape and distribution of pores in their three-dimensional matrix [38]. The capacity of water absorption and the swelling kinetics of the hydrogel depend directly on the pore architecture, the presence of hydrophilic functional groups and the elasticity of the network. [13] Thus, a network with regular distributed pores which have tunable shape and dimension will allow a better attachment of cells, with good application in 3D cell culture. As seen in the SEM images (Figure 9), the co-assembled gels have a homogeneous structure with interconnected pores. The fragile structure and smooth surface of gels are independent on the ratio between the compounds. However, the increase of FMOC–Trp–OH content in the hydrogel composition leads to a denser and less homogeneous structure.

## 3. Conclusions

The study presents the preparation of new physical hydrogels based on FMOC amino acids. The volumetric ratio between the two compounds influences the stability and the final strength (given by G′ value) of the obtained hydrogels. In the case of FMOC–Trp–OH stock solution, it was used a pH-switch approach while for FMOC–Lys–FMOC–OH stock solution was used a polar solvent. The co-assembly of the amino acids in different volumetric ratios generated stable structures with transparent aspect and solid-like behavior evidenced in oscillatory shear tests. The stability and transparent aspect are influenced by the composition of the hydrogels. By increasing the amount of FMOC–LysFMOC–OH, stable hydrogels are obtained, but with a slightly translucent aspect, while by increasing of the FMOC–Trp–OH ratio transparent weak hydrogels are attained. The FT-IR spectra emphasized the co-assembly of the FMOC amino acids through the intra and intermolecular bonds formation. SEM microscopy supports the reorganization at the molecular level and the obtainment of the three-dimensional structures. The UV-Vis, fluorescence and circular dichroism spectroscopy results show that supramolecular co-assembly process was induced by π–π overlapping of fluorenyl moieties.

## 4. Materials and Methods

### 4.1. Materials

N–(9–Fluorenylmethoxycarbonyl)–Tryptophan–OH amino acid (FMOC–Trp–OH, C26H22N2O4, Mw = 426.46 g/mol, purchased from Novabiochem^®^ (Germany)) and FMOC–Lys–FMOC–OH (C_36_H_34_N_2_O_6_, Mw = 590.66 g/mol, from Sigma-Aldrich (Darmstadt, Germany)) amino acids were used as received. Sodium phosphate buffer (PBS, pH 7.4, 0.1M) was prepared using monosodium phosphate (NaH_2_PO_4_ × 2H_2_O) and disodium phosphate (Na_2_H_2_PO_4_ × 7H_2_O) in distilled water via standard protocol. Citric acid was acquired from Sigma-Aldrich (Darmstadt, Germany), dimethyl sulfoxide (DMSO) from Fluka (Buchs, Switzerland).

### 4.2. Method

The formation of the hydrogels from FMOC–Trp–OH and FMOC–Lys–FMOC–OH was realized by individually preparing of the two stock solutions and by mixing them in different volumetric ratios to obtain three different variants.

For the preparation of FMOC–Trp–OH solution, pH change technique was used for gelling triggering. Briefly, 0.5% FMOC–Trp–OH was dissolved in 0.05 M NaOH. The mixture was stirred for a few minutes until the solution became transparent. After complete dissolution of the powder a few drop-wise of 0.1 M citric acid (~20 µL) were added until the pH of the solution was stabilized at around pH 7.4. Further, 0.1 M phosphate buffer solution (pH = 7) was added and the solution was gently stirred with a spatula until the mixture became translucent.

In the case of preparing the FMOC–Lys–FMOC–OH solution, the approached technique was the use of a polar solvent. Hence, 0.5% FMOC–Lys–FMOC–OH was dissolved in dimethyl sulfoxide (DMSO) and then mixed with a spatula until the solution became transparent. Over the transparent solution was gradually added 0.1 M phosphate buffer solution (pH = 7) to avoid the formation of a film on contact between the two solutions. Initially, the solution became opaque, but turned into a transparent gel after about 3 min.

After the individual preparation of the stock solutions, three variants of co-assembled hydrogels were obtained. The difference between the three variants was given by the volumetric ratio between the amino acid solutions, namely FMOC–Trp_FMOC–Ly–FMOC in 1/1 ratio, FMOC–Trp_FMOC–Lys–FMOC in 1/3 ratio, and FMOC–Trp_FMOC–Lys–FMOC in 3/1 ratio. Practically, FMOC–Trp–OH solution was added over FMOC–Lys–FMOC–OH solution, the mixture was slightly homogenized and left at room temperature for 24 h. In all three cases, stable gels were obtained that passed the vial inversion test (Figure 7).

The co-assembly of the gels was done after pH change and water addition in the individual compounds. FMOC–Trp and FMOC–Lys–FMOC were mixed in different ratios (Table 3).

The gels obtained through co-assembly processes between the two functional amino acids with an aromatic group (FMOC) on the N–terminal had a stable structure confirmed by the vial inversion test (Figure 7). Additionally, self-supporting gels had a transparent aspect following the co-assembly process.

The FMOC–Trp–OH_FMOC–Lys–FMOC sample in 1/3 ratio presented viscoelastic behavior and had a translucent aspect, while the FMOC–Trp–OH_FMOC–Lys–FMOC sample in 3/1 ratio was slightly unstable, which was attributed to the lower amount of lysine. Subsequently, the prepared hydrogels were lyophilized for FTIR and SEM characterization. The other analyses (fluorescence, circular dichroism, UV-Vis, rheology) were realized in gel state. DLS measurements were made in solution (before gelification) in order to evaluate the stability and the interactions which occur between the components.

### 4.3. Characterization

#### 4.3.1. Dynamic Light Scattering (DLS) Measurements

DLS measurements of the particles of the self-assembled gels based on amino acids were performed using a Malvern Zetasizer Nano ZS instrument (Worcestershire, United Kingdom) equipped with a 4.0 mW He–Ne laser operating at 173 °C (backscatter mode) on 633 nm. Particle size distribution and the polydispersity index (PDI) of the gel particles, as well as the Zeta potential measurements, were determined. The hydrodynamic diameters (DH) of the gel particles were calculated from diffusion coefficients using the Stokes-Einstein equation.

#### 4.3.2. UV-Vis Absorption

Optical properties of hydrogels were obtained by using UV-Vis reflectance spectra measured at an Analytik Jena SPECORD UV/Vis 210Plus spectrophotometer.

#### 4.3.3. Fluorescence Measurements

The fluorescence emission spectra of gels were recorded using a Perkin Elmer fluorescence spectrophotometer. The excitation wavelength was 285 nm and the emission and excitation slits width were set at 6 and 3 nm. The fluorescence spectra were obtained in the range of 290–550 nm at an integration time of 1.0 s/nm.

#### 4.3.4. Circular Dichroism Measurements

The circular dichroism (CD) spectra were measured using a Chirascan plus (Applied Photophysics) spectrometer by using 2 mm path lamellar cells. The CD spectra were recorded from 400 to 230 nm a with step size of 1 nm and bandwidth of 1 nm. The measurements were performed at room temperature (22 °C).

#### 4.3.5. Structural Characterization

Fourier transform infrared spectra of the lyophilized gels were recorded from KBr pellets with Vertex 70 spectrophotometer Bruker-Germany). The vibrational transition frequencies were reported in wavenumbers (cm^−1^) and the FT−IR spectra were recorded on 400–4000 cm^−1^ interval, at 4 cm^−1^ resolution.

#### 4.3.6. TG/DTG Analysis

The thermal stability of the lyophilized gels was evaluated by thermogravimetric analysis on a STA 449 F1 Jupiter apparatus (Netzsch-Germany). The samples, with weights from 8 to 10 mg, were placed in an open Al_2_O_3_ crucible and thermally degraded. The temperature range was 30–670 °C in N_2_ (99.99% purity) atmosphere. Runs were performed in a dynamic mode, with a heating rate of 10 °C/min under a gas flow of 40 mL/min. The thermogravimetric balance was calibrated on temperature and sensitivity with standard metals (In, Sn, Bi, Zn and Al) from 25 to 700 °C. Data collection was performed with Proteus^®^ software

#### 4.3.7. Rheological Investigation

The viscoelastic characterization was done by using MCR 302 Anton Paar rheometer (Gratz, Austria) equipped with plane-plane geometry (the diameter of the upper plate being 50 mm, gap of 500 μm) and with Peltier device for temperature control.

The viscoelastic parameters were determined in oscillatory shear conditions. The elastic modulus, G′, quantifies the stored deformation energy; the viscous modulus, G″, is a measure of the dissipated energy and the loss tangent, tan δ, expresses the degree of viscoelasticity as the ratio between the loss energy and the accumulated energy during a cyclic deformation. The dynamic oscillatory behavior of the samples was investigated at 25 °C in frequency sweep tests in the linear range of viscoelasticity (γ = 0.3%). The linear domain of viscoelastic behavior was determined for each sample in amplitude sweep tests at an oscillation frequency of 1 rad/s.

#### 4.3.8. Morphology Analysis

Preparation of the samples for morphological characterization by SEM investigations was made by freezing the preformed gels with liquid nitrogen until all the water was sublimated. SEM investigations were performed on samples fixed in advance by means of colloidal copper supports. The samples were spray−coated with a thin layer of gold (K Emitech550X). The area covered was examined by using a scanning electron microscope type Quanta 200, which operates at 30 kV with secondary electrons in high vacuum mode.

## Figures and Tables

**Table 1:** The registered frequencies [cm^−1^] in FT-IR spectra of the freeze-dried gels and their absorption assignments.

	FMOC_Lys_FMOC–OH	FMOC–Trp_FMOC–Lys–FMOC: 1/3	FMOC–Trp_FMOC–Lys–FMOC: 1/1	FMOC–Trp_FMOC–Lys–FMOC: 3/1	FMOC–Trp_OH
N–H 2 peaks primary amine/ 1 peak secondary amine	3343 and 3379	3424 and 3329	3418 and 3327	3418	3426 and 3347
C–H alkane	3046	3057	3055	3055	3046
C–H stretching Aromatic ring	2947	-	2940	2942	2951
C=O as stretching amide I band	1689	1695	1703	1705	1736
C–N Amide II band	1531	1535	1533	1522	1520
CH2	1448	1446	1446	1443	1444
C–Namide III band	1250	1257	1253	1252	1219
C–O stretching	-	1155	1161	1169	-
C–O	1062	1076	1068	1068	1091
Aromatic ring					
C=C C–C C–H	945 864 736	940 860 742	947 858 744	947 860 744	- 879 748

**Table 2:** Parameters of the thermal decomposition of freeze-dried gels.

Sample	Degradation Stage	T_onset_ (°C)	T_peak_ (°C)	T_end_ (°C))	W (%)	Residue	T_10_ (°C)	T_20_ (°C)	Tg (°C)
FMOC_Trp_FMOC–Lys–FMOC: 1/3	I	157 193 317	161	184 282 356	11.98	32.51	171	202	
	II		204		34.98				34.5
	III		329		20.53				
FMOC–Trp_FMOC–Lys–FMOC: 1/1	I	105	140	167	9.59	29.48	169	196	
	II	183	197	205	17.12				30.1
	III	263	291	305	24.40				
	IV	315	332	368	19.41				
FMOC–Trp_FMOC–Lys–FMOC: 3/1	I	136	163	174	20.06	27.69	152	174	
	II	171	203	224	12.41				48
	III	216	282	295	12.29				
	IV	290	329	370	27.55				

T_onset_—the temperature at which the thermal decomposition begins; T_peak_—the temperature at which the degradation rate is maximum; T_endset_—the temperature at the end of the process; W—weight losses; T_10_ and T_20_—the temperatures corresponding to 10 and 20 wt.% weight losses.

**Table 3:** Samples name and method of preparation.

Sample	Composition for a Volume of 5 mL Sample
FMOC–Trp_FMOC–Lys–FMOC: 1/3	1.25 mL FMOC–Trp–OH added over 3.75 mL FMOC–Lys–FMOC–OH
FMOC–Trp_FMOC–Lys–FMOC: 1/1	2.5 mL FMOC–Trp–OH added over 2.5 mL FMOC–Lys–FMOC–OH
FMOC–Trp_FMOC–Lys–FMOC: 3/1	3.75 mL FMOC–Trp–OH added over 1.25 mL FMOC–Lys–FMOC–OH

## Data Availability

Not applicable.
